# “Only One Way Out”-Partners' Experiences and Grief Related to the Death of Their Loved One by Suicide or Physician-Assisted Dying Due to a Mental Disorder

**DOI:** 10.3389/fpsyt.2022.894417

**Published:** 2022-07-08

**Authors:** Marianne C. Snijdewind, Jos de Keijser, Gerty Casteelen, Paul A. Boelen, Geert E. Smid

**Affiliations:** ^1^ARQ Centrum'45, Diemen, Netherlands; ^2^Department of Ethics, Law and Humanities, Amsterdam UMC, Academic Medical Centre, Amsterdam, Netherlands; ^3^Department of Clinical Psychology and Experimental Psychopathology, Faculty of Behavioral and Social Sciences, University of Groningen, Groningen, Netherlands; ^4^Expertisecentrum Euthanasie, Den Haag, Netherlands; ^5^ARQ National Psychotrauma Centre, Diemen, Netherlands; ^6^Department of Clinical Psychology, Faculty of Social Sciences, Utrecht University, Utrecht, Netherlands; ^7^Department of Humanistic Chaplaincy Studies, University of Humanistic Studies, Utrecht, Netherlands

**Keywords:** grief/loss, suicide, physician-assisted dying (PAD), mental disorder, mental health

## Abstract

**Background:**

Previous research has provided insight into the grief of suicide survivors, but little is known about grief following physician-assisted dying (PAD), and no prior study specifically focused on grief following PAD due to a mental disorder. The current study aims to increase insight into experiences preceding PAD or suicide of a loved one due to a mental disorder and their impact on mental health symptoms.

**Methods:**

We performed a survey study and in-depth interviews with 27 bereaved life partners. The deceased had been in treatment for mental disorders and had died by PAD (*n* = 12) or suicide (*n* = 15). Interviews explored grief experiences and experiences with mental health care. In the survey, we assessed self-reported symptoms of grief, post-traumatic stress, anxiety, depression, quality of life, and impairments in social, and occupational functioning.

**Results:**

All participants reported generally low levels of mental health symptoms. Longer time since death and death by PAD were associated with lower grief intensity. Interviews showed various degrees of expectedness of the partners**'** death, and a varying impact of being present at the death on bereaved partners.

**Conclusion:**

Expectedness of the death of the partner, absence of suffering of the partner at the time of dying, and presence of physician support may in part explain the protective effects of PAD against severe grief reactions. Physicians considering their position regarding their personal involvement in PAD due to a mental disorder could take grief reactions of the bereaved partner into account.

## Introduction

Grief following the loss of a loved one is influenced by the way in which the loved one died. Following death by an unnatural cause, grief reactions in the bereaved survivors are likely to be more severe than following death by a natural cause (1). Suicide is one of the causes of unnatural death. About 1 in 5 people are exposed to the suicide by a family member, friend, classmate or other acquaintance in their life (2), and bereaved relatives after suicide are at increased risk of mental health problems (3, 4), e.g., post-traumatic stress disorder symptoms (5–7), depressive symptoms (4), impairments in functioning (3), suicidal behavior, and suicide (3, 4). Suicide death may be violent, i.e., mutilating (e.g., strangulation or high impact collision) or non-violent, i.e., non-mutilating (e.g., physician-assisted dying (PAD), drug overdose, ingestion of a deadly substance, helium inhalation). Violent deaths are more likely to generate distressing intrusive memories in the bereaved than non-violent deaths (8). Yearly, over 700.000 people die by suicide worldwide −1.3% of all deaths in 2019 (9). In the Netherlands in 2020, 1,823 people died by suicide (1.1% of all deaths) (10), suicide being due to a mental disorder in 1,035 (56.8%) cases (11).

Grief following the loss of a loved one is strongly related to the meaning the bereaved person attributes to the loss, which in turn is determined by individual, social, and cultural factors, the circumstances of the death, and the relationship to the deceased (12). Thus, grief in suicide-bereaved people is influenced by the death of the loved one being caused by an intentional act. Suicide, however, is not the only cause of death crucially depending on an intentional act of the person who dies, as this is also the case in physician-assisted dying (PAD). Nevertheless, this latter group seems to be overlooked in current research.

Physician-assisted dying, which comprises euthanasia (wherein the physician administers the euthanatic) and physician-assisted suicide (wherein the physician provides the euthanatic), is legally regulated in a few countries worldwide (13). In the Netherlands, physicians providing assistance in dying are exempted from criminal liability if they fulfill the following statutory due care criteria: the physician must be convinced that the patient is making a voluntary and well-considered request and that the patient's suffering is irremediable and unbearable; the physician and the patient must be convinced that there is no reasonable alternative to the situation; at least one other physician must see the patient and provide a written opinion on the above criteria; the assisted death or euthanasia must be performed in a medically prudent manner (14). These legal criteria encompass PAD due to a mental disorder. While in 2021 a total of 7,666 people died by PAD in the Netherlands (4.5% of total deaths), only 115 of these (1.5% of all reported PAD-cases) concerned PAD due to a mental disorder (15), 0.07% of total deaths (10).

A previous study into the perspective of physicians on ending one's own life due to a mental disorder indicated a continuum ranging from impulsive suicidality to a well-considered and persistent wish to die (16). PAD often cannot be considered an alternative for suicide, because of patient characteristics or legal restrictions. Indeed, not all people who die by suicide requested PAD prior to the suicide. Although suicide does occur after requesting PAD and some patients continue to request PAD following a refused request for PAD (17–20), PAD is only a valid option if the due care criteria are met.

However, in some situations PAD may be considered a potential alternative for suicide. Some people suffering from a mental disorder die by suicide while on the waitlist for a PAD request (17, 21). Disapproval of the option of PAD by family members can cause a patient to commit suicide even after the PAD-request was approved by the physicians involved (21). Furthermore, some people with mental disorders seem to be afraid to request PAD from their physician because they fear this will put them in compulsory admission (22). They presumably would request PAD if their relationship with their physician would improve and their fear of a compulsory admission would be allayed. In addition, more than half of physicians find it inconceivable to perform PAD in psychiatric patients (23) and individual interpretations and considerations weigh in when a request for PAD is considered (24). Thus, restrictions regarding the system of care and the caregiver attitude may hinder people who could be eligible to have a request for PAD granted, highlighting situations in which PAD could be an alternative to suicide.

Research on grief following PAD, though scarce, indicates that PAD-bereaved people experience feelings of isolation and fear of social stigma, but knowing PAD was an autonomous choice helps them accept the death (25–28). Indeed, PAD-bereaved showed less symptoms of grief and post-traumatic stress disorder (PTSD) than bereaved who had experienced a natural loss (25–27). To the best of our knowledge, no previous study focused specifically on grief after PAD due to a mental disorder.

The current study aims to clarify how circumstances or experiences during the period leading up to the PAD or suicide of a loved one due to a mental disorder impacts the experienced grief, associated mental health symptoms, and functioning of the bereaved partners. For this, we used mixed methods to conduct an exploratory qualitative semi-structured in-depth interview study combined with a comparative quantitative survey.

## Materials and Methods

We consulted the Medical Ethics Research Committee Utrecht (protocol number 19/596), who exempted the study from formal review because the Medical Research Involving Human Subjects Act (WMO) does not apply to the study.

### Participants

The inclusion criteria for this study were: (1) loss of a partner by PAD or suicide due to mental disorder, (2) time elapsed since the loss between 6 months and 10 years, and (3) the partner had been on a waiting list for or had received mental health treatment for at least 2 years prior to the death. Participants were recruited through Expertisecentrum Euthanasie (Euthanasia Expertise Centre, The Netherlands). In the Netherlands, most of the cases of PAD due to a mental disorder are performed by physicians working for Expertisecentrum Euthanasie (15). Expertisecentrum Euthanasie selected all cases of PAD in patients with mental disorder where a life partner had been involved and contact information was available. These partners received an information letter about the research, including a response card. One reminder was sent. A total of 21 potential participants were invited: we received 10 reactions of people who were willing to participate. All met the inclusion criteria and were included in the research. One response card was returned indicating a refusal.

Next, we used (social) media to recruit participants. An invitation, placed on the website of ARQ Centrum'45, was distributed through social media by the University for Humanistic Studies and 113 Suicide Prevention. During a symposium for suicide survivors, held on December 11th 2020, the attendees were informed about the research and the possibility to participate. A newsletter of the Friends of Expertisecentrum Euthanasie contained information about the research and a call to participate. Through these media, 24 people responded, of which 10 were eligible. We also used snowball sampling to recruit participants, by sending out flyers about the research in our professional network and actively asking people to participate. Nine people responded, of which seven met the inclusion criteria.

### Procedure

The study combined a quantitative survey with a qualitative interview. Two weeks prior to the interview, participants were mailed the survey that included background and loss-related questions and self-report questionnaires to assess grief reactions, PTSD symptoms, anxiety and depression, general quality of life, and functional impairment.

Traumatic Grief Inventory-Self-report Plus (TGI-SR+). The TGI-SR+ (29) consists of 22 items to assess the intensity of grief reactions rated on a 5-point Likert scale. Grief severity can be calculated as a sum score and according to criteria sets for Persistent Complex Bereavement Disorder (PCBD) as per DSM-5, and Prolonged Grief Disorder (PGD) as per ICD-11 and DSM-5-TR (results of the analyses using DSM-5 PCBD, DSM-5-TR PGD, and ICD-11 PGD as outcomes are presented as Supplementary Tables 2, 3). A total score ≥71 indicates probable PCBD/PGD. The internal consistency (Cronbach's alpha) in the current sample was 0.94.

Post-traumatic Stress Disorder Checklist for DSM-5 (PCL-5) (30), Dutch version (31). The PCL-5 consists of 20 items to measure the severity of the PTSD symptoms rated on a 5-point Likert scale. A total score ≥33 indicates probable PTSD (15, 16). Cronbach's alpha was 0.86.

Hospital Anxiety and Depression Scale (HADS) (32), Dutch translation (33). The HADS includes a depression subscale as well as an anxiety subscale, each consisting of seven items rated on a 4-point Likert scale. A score ≥8 on each subscale indicates probable depression or anxiety disorder (34). Cronbach's alpha was 0.92.

World Health Organization Quality Of Life-BREF (WHOQOL-BREF) (35) indicates the respondent's perception of his or her quality of life rated on a 5-point Likert scale. To minimize participants' time investment, we only used the first question, assessing general quality of life. Scores were transformed to a score between 0 and 100.

Work and social adjustment scale (WSAS). A grief adapted version of the WSAS (36) assesses functional impairment because of the loss, rated on a 9-point Likert scale. A total score ≥21 indicates probable impairment (36). Cronbach's alpha was 0.81.

The qualitative interviews were all conducted by a researcher with extensive interview experience. Participants were informed about the purpose of the study and provided written or verbal, recorded, consent. Interviews were semi-structured, using a topic list (see Supplementary Table 1). All interviews were held between February 2020 and March 2021, face-to-face at participants' homes, except three videoconferencing interviews. Interviews lasted between 46 and 231 min and were all recorded and transcribed.

### Statistical Analyses

Survey data analyses were performed using SPSS version 27.0 for Windows. Missing scale item responses were present in a mean of 0.5% of responses per case (range, 0 −3.6%) and were handled using mean imputation. Prior to the final analyses, we checked normality by verifying that none of the variables had skewness or kurtosis values smaller than −3 or larger than 3. Descriptive statistics were calculated for the complete sample. Chi-square tests or Fischer exact tests and independent *t*-tests were conducted to examine sociodemographic and mental health related differences between the PAD and suicide-bereaved partners. Regression analyses evaluated the impact of mode of partner death and time since death on mental health related variables with and without adjustment for the effects of other loss-related variables, specifically, being present at the death and violent death. To enable this, independent variables were entered to the regression in two steps, and the significance of the *R*^2^ change was calculated for the second step. Visual inspection of residual plots confirmed homoscedasticity. There was no evidence of multicollinearity, as indicated by tolerance values >0.25. Alpha level was set at 0.05 for statistical significance.

### Qualitative Analyses

By inductive coding, we conducted a thematic analysis using ATLAS.ti (37). The first three interviews were open coded by three researchers and these codes were extensively compared and discussed until an agreement was reached. Based on this, a coding scheme was drafted that was further developed through coding of the next seven interviews. Thereafter, codes were frequently grouped and regrouped into overarching themes. Codes were added when new information emerged during analyses. Data analysis was frequently discussed between the authors and agreement was reached by discussing the interpretation of specific quotations in the context of the entire interview.

## Results

### Sample Characteristics

Twenty-seven participants were included in this study, of which twelve had experienced the death of their partner by PAD and fifteen by suicide. We divided the cause of death into violent, i.e., mutilating, and non-violent, if the body was not mutilated. All PAD-bereaved participants had been present at the death. Of the suicide-bereaved participants, some had been present at the death, if the suicide was non-violent. Table 1 details causes of partner's death and participants' presence at the death.

**Table 1:** Causes of partner's death and respondents' presence at the death.

**Cause of partner's death**	***N***	**Respondent present**	
		***N***	**%**
**Suicide**	15	3	20.0
**Violent causes**	8	0	0.0
Strangulation	4	0	0.0
Being hit by an object at high speed	3	0	0.0
Jumped from a high building	1	0	0.0
**Non-violent causes**	7	3	43.0
Ingesting a deadly substance	3	2	66.7
Inhaling a deadly substance	2	1	50.0
Medication overdose	2	0	0.0
**PAD**	12	12	100.0
**Non-violent causes**	12	12	100.0
Euthanasia	8	8	100.0
Physician-assisted suicide	4	4	100.0

Table 2 lists the characteristics of the participants and their partner's death and mental health outcomes. Suicide-bereaved participants were higher educated and more often female than PAD-bereaved participants. Suicide-bereaved participants and their partners were significantly younger, and the duration of the relationship was shorter, and more time had passed since the death compared to PAD-bereaved participants. Mean scores on mental health outcome scales indicated generally low levels of distress, with mean scores well below cut-off scores suggesting clinically relevant levels of distress or impairments in functioning. Mean perceived general quality of life was between good and neither poor nor good.

**Table 2:** Sociodemographic variables and mental health outcomes by mode of partner's death.

	**Full sample**		**Mode of partner's death**				
	**(*****N*** **= 27)**		**Suicide (*****N*** **= 15)**		**PAD (*****N*** **= 12)**		**χ^2^ (df = 1)**
	***N***	**%**	***N***	**%**	***N***	**%**	
**Gender**							
Female	13	48.1	10	66.7	3	25.0	4.64^*^
Male	14	51.9	5	33.3	9	75.0	
Higher education	17	63.0	14	93.3	3	25.0	13.35^***^
Children	20	74.1	12	80.0	8	66.7	0.62
Filed a complaint	2	7.4	2	13.3	0	0.0	1.73
Present at partner's death	15	55.6	3	20.0	12	100.0	17.28^***^
Violent partner's death	8	29.6	8	53.3	0	0.0	9.10^**^
	**M**	**SD**	**M**	**SD**	**M**	**SD**	***t*** **(df = 25)**
Age	61.02	13.11	54.27	11.45	69.46	9.99	−3.62^**^
Years since partner's death	2.63	2.52	3.52	2.81	1.51	1.59	2.21^*^
Age partner	58.15	14.61	49.83	11.03	68.56	11.71	−4.27^***^
Duration of relationship	30.00	18.22	21.20	14.81	42.04	15.58	−3.55^**^
Grief reactions	44.72	14.82	47.40	13.94	41.37	15.82	1.05
PTSD symptoms	8.80	8.24	10.22	8.35	7.02	8.09	1.00
Anxiety symptoms	4.04	2.92	5.00	2.42	2.83	3.13	2.03
Depression symptoms	3.84	3.65	4.20	3.93	3.39	3.38	0.57
General quality of life	77.78	14.01	77.50	15.81	78.13	12.07	−0.11
Functional impairment	8.08	7.70	9.65	8.27	6.13	6.74	1.19

* *P < 0.05*.

** *P < 0.01*.

*** *P < 0.001*.

The following paragraphs describe the outcomes of the interviews, in which the respondents described the relationship with their partner and the period leading up to the death of their partner, focusing on the impact of partner's mental health problems, anticipating partner's death, experiences of professional care, and being present at partner's death.

### Impact of Partner's Mental Health Problems

All respondents recalled when they first noticed their partner was suffering from mental health problems. The impact of the mental disorder on the lives of the respondents ranged from very little when the disorder was only manifest during limited periods, to very large when the disorder was ever present and/or the partner attempted suicide several times. The perceived burden this placed on the respondents varied. Many respondents recognized only in hindsight how the disease of their partner had affected them (R9, Table 3). Respondents never seriously considered to leave their partner, even if this was suggested by friends or family. Some mentioned the bond with their partner remained strong and the relationship was still reciprocal, others just didn't want to let their partner down (R9, Table 3).

**Table 3:** Impact of partner's mental health problems.

R9, violent suicide, not present	*In retrospect, only when you take a step back, do you realize how deeply it affects you. (…) I thought: I want to grow old with you. So I also thought, like, yeah, you don*'*t just abandon someone if you have these feelings. You don*'*t say, “okay you*'*re in a rough spot, goodbye, take care”. You go way too far, of course. However, in hindsight I*'*m very glad I did. Now I can say I did everything I could. And even then, the moment he passed away, I felt a sense of guilt*.
R11, non-violent suicide, not present	*In the evening he would self-mutilate. Not in my presence, because he knew I didn*'*t like that. He did it at night. But I also have two children. I said I don*'*t want anything to happen here and risk that my children, or I, find you in the morning, here in the house. Because I have to live on, and I think I would suffer from that the rest of my life. We then decided we would live separately*.
R23, PAD, present	*When she had those stabs, the neighbor, who was working in the garden, heard screaming. He asked what happened to Elsa. [I replied] How come? [He responded] Yeah, she screamed this morning. I said: she*'*s in the hospital right now. Yeah, he said, I saw the ambulance. I said, yes, she wanted to end her life. But it failed, I said*.

*Quotes are preceded by the respondent code, mode of the partner's death, and whether the repondent had been present during the partner's death*.

For some participants, periods when their partner was hospitalized meant rest and time to recover, for others–most often respondents with children–hospitalization of the partner just added other tasks, such as visiting the care institution and taking care of the children, to an already overflowing schedule. Respondents tried to shield their children from (self-) destructive expressions of the disorder. Some couples with children decided to live apart when manifestations of the disorder might negatively affect their children (R11, Table 3). The impact of partner's mental health problems on PAD- and suicide-bereaved respondents of partners appeared similar and several participants from both groups had experiences with previous suicide attempts (R23, Table 3).

### Anticipating Partner's Death

For many suicide-bereaved respondents, their partner's death was to some extent expected. For some, this started while talking about the struggle their partner felt to continue living, others were confronted with a suicide attempt. Conversations about ending one's life were difficult but valuable. Hearing one's partner was no longer able to continue living was for most respondents not something they could immediately understand, let alone accept, but having conversations on this topic made the eventual death more comprehensible. One respondent clearly explained this process toward acceptance (R1, Table 4). The conversations could also be the starting point to try and find better therapy or medication. Conversations included discussions about the preferred means to die by. The preference to die by PAD was often expressed, but partners then had to find a physician willing to help them. This proved to be an insurmountable obstacle for some partners, who could not find a willing physician, were told that the due care criteria were not met, or for whom the waiting time was too long. For these partners, this ultimately resulted in dying by suicide. Conversely, one respondent's partner initially preferred to die by a non-violent, planned suicide, but did not want to burden the respondent with a police investigation afterwards, and therefore pursued a PAD-request with success (R6, Table 4).

**Table 4:** Anticipating partner's death.

R1, PAD, present	*I think, before telling me, she thought about it herself. For a long time, she had hope that there was something that could help her. That*'*s what actually kept her going, hope. (…) It was not going well and then she was admitted for a while. And then I kind of had the idea, yes, it [PAD] might happen, it might actually take place. So then I started to get used to the idea myself. It is, yes, it is something that you have to get used to, too. It is, yeah, it is weird so to speak. And in the end, it's not something, yeah, that you say like “oh, well then we*'*ll do that, right?” You know. That's not how it works. Yes, the idea slowly sinks in. I: And how does that happen? R: Well, by talking about it, just the two of us. And also, with others, I did talk about it. For example, at work I have told my supervisor. (...) By talking about it with others, you also come to the conclusion for yourself that it could be, yes, that it*'*s the only option, so to speak. And yes, it's not a solution, but it*'*s more of a way out, so to speak. (…) Because it has been such a long process so to speak, the idea slowly grows on you. And I think six months in advance I already had an idea that it*'*s alright, so you accept it. And yes, I think that helps you too, or at least it helped me, move on*.
R6, PAD, present	*She didn*'*t want to put me in trouble. (…) Be prepared the police will show up on your doorstep. If we would do it ourselves, and she dies, you won*'*t get the normal route where the doctor just signs. They can even arrest you. (..) Of course you can convince the police and judiciary what has happened, show them the euthanasia request, a written letter, status. Yes, we could*'*ve done that. But she wanted to relieve us from all that*.
R5, non-violent suicide, present	*I: How did you feel about the date being set in advance? R: Yeah, well, it's alright, because you know where you stand–in the sense that it won*'*t just happen tomorrow. It*'*s something you can live towards. But it is also a threatening monster that slowly comes towards you. In that sense it is very twofold*.

*Quotes are preceded by the respondent code, mode of the partner's death, and whether the repondent had been present during the partner's death*.

Planning the date for the death occurred both in PAD and suicide. For some respondents, this was uncomfortable, as they felt that the bond between them and their partner waned as their partner was withdrawing from connections with life. One respondent felt the partner living toward death, that was dreaded by the respondent (R5, Table 4). Suicide-bereaved respondents who had been present at their partner's death had all been involved in deciding about the method of suicide and seeking information on humane means of suicide. They knew that being present during the suicide would entail being considered a potential suspect by the police, since assisting in suicide is considered a crime. One respondent recorded the suicide on video to document absence of assistance.

### Experiences of Professional Care Related to a Wish to Die

Suicide-bereaved respondents who did not anticipate the death of their partner mentioned that in a professional care setting their partner had denied having suicidal thoughts or tendencies. These respondents explained the suicide had happened in a state of severe distress, that their partner had not meant to die and that their death should have been prevented (R8, Table 5). Other respondents were not so outspoken about the possibility and desirability of preventing the death of their partner. Respecting and understanding the feeling that life was no longer livable did not mean that these respondents had lost hope that their partner in the end would stop pursuing PAD or suicide, or that positive changes would occur regarding their partner's mental health disorder (R25, Table 5).

**Table 5:** Experiences of professional care related to a wish to die.

R8, violent suicide, not present	*I think she laid awake that last night, and that she went into psychosis. That*'*s what I think. And that*'*s why I didn*'*t see it coming (cries). If you do something like that, when you look into it… You*'*re in a state of absolute tunnel vision. Yeah, then you*'*re not normal. So I guess, I can never figure it out, but this is what I think happened: she went into psychosis, and thought this was the solution. Yeah, clearly it wasn*'*t, but okay*.
R25, non-violent suicide, not present	*She made it very clear she didn*'*t see any way out, didn*'*t see a way back. It was a decision she took. So all hope was lost. But still, you keep hoping something happens. But with every attempt she took… You*'*re almost glad that she succeeded at some point. (…) It is often an act of desperation, because there is no other way out. If care is good and all options are on the table, and someone then chooses they don*'*t want to live any longer… Yeah, preferably by a humane method of course, by means of euthanasia for example. But those options must be there. Anne didn*'*t have any option*.
R14, violent suicide, not present	*They were all very reluctant. Mainly damage control. That made me very angry. I: What were your expectations of that conversation? I can*'*t recall exactly. Of course I was very angry and frustrated that it [the suicide] could*'*ve happened there. On the other hand, I felt they hadn*'*t made a mistake on purpose or anything. (…) It was not my intention to file all kinds of complaints, or hang people from the highest tree. But, when I went there, I did, because there was just so little understanding. There was no recognition. There was no compassion. It was mainly like* '*yes we are very sorry; and then it was like “well, we did this, we did it like this and we did what we could and we can't be blamed*”*, that*'*s the impression I got. So I walked away really angry. (…) She [the psychiatrist] stroke a completely different tone. She knew what to do, and she immediately took the sting out of the situation, she was very empathic. For me, the anger was almost gone afterwards*.

*Quotes are preceded by the respondent code, mode of the partner's death, and whether the repondent had been present during the partner's death*.

Many respondents were dissatisfied by the professional care that their partner received. They signaled lack of care providers' recognition of the severity of their partner's suffering, care providers not listening to respondents while they explained the severity of their partner's suffering, physician's unwillingness to consider a request for PAD, and long duration of the partner's suffering before a PAD request was granted. Several respondents trying to find answers from care providers to questions after their partner's death were left empty-handed due to privacy regulations or care provider's fear of being sued. This led to frustration for the respondents, who felt they needed answers to move on or make sense of what happened. An emphatic reaction could take this frustration away (R14, Table 5).

### Being Present at the Death

A respondent who was present while the partner ingested a deadly substance described the situation as aggressive and damaging for herself to have witnessed, as the partner struggled to find the correct way to take in the deadly means and the partner's body put up resistance against death (R5, Table 6). Respondents who had not been present at their partner's non-violent death often felt bad about this, although this had most often not been a conscious decision. They had wanted to support their partner and regretted the lonely death. Even if not present, some respondents more or less knew the moment their partner would die by suicide and had to decide whether or not to intervene. After having prevented several suicide attempts, two respondents decided not to intervene during the final attempt. For one of them, this was prompted by the idea that it was legally not allowed to be present during the suicide. Being absent subsequently led to a feeling of guilt and a feeling of having failed as partner (R11, Table 6). The other respondent was convinced this was what the partner wanted and was glad to set the partner free.

**Table 6:** Being present at the death.

R5, non-violent suicide, present	*It was like an intermediate method: planned like euthanasia, but a suicide, like a suicide with an aggression to it, or a force that is needed to do that. It was so powerless [for me], it was, well, a struggle sitting there. Wanting to be there for her, but basically everything in me wanted to leave the room. I wanted to support her in the way she wanted, while I also felt a hard “no” from within: don*'*t do this, stop! It*'*s lacerating*.
R11, non-violent suicide, not present	*The thought that he, so consciously and alone, in solitude... Yeah, I would*'*ve rather be–that would have been difficult too–but I*'*d rather been present. But that*'*s not allowed, he knew that as well. How lonely is that? I: You talked about that, too? Very often. But it was not allowed. And he knew that too. Yes, we talked about it a lot*.

*Quotes are preceded by the respondent code, mode of the partner's death, and whether the repondent had been present during the partner's death*.

### Survey Results: Mental Health Outcomes

The results of the analyses of the survey data are shown in Table 7. Both time since death and death by PAD (vs. suicide) were associated with lower grief reactions. When adding violent death and being present at the death as independent variables (step 2), time since death and death by PAD were still negatively associated with grief reactions. Years since death and death by PAD were not independently associated with anxiety, depression, general quality of life, or functional impairment. However, when adding violent death and present at death as independent factors (step 2), death by PAD was associated with less anxiety and depression.

**Table 7:** Multiple regressions predicting mental health outcomes based on mode of partner's death and time since death.

	**Step 1**						**Step 2**							
	**B**	**95% CI**		**Beta**	**F**	***R*** ^**2**^	**B**	**95% CI**		**Beta**	***F***	***R*** ^**2**^	***ΔR*** ^**2**^	**ΔF**
**Grief reactions**														
Years since death	−3.48	−5.69	−1.27	−0.59^**^	6.04^*^	0.33	−3.67	−5.80	−1.53	−0.62^**^	4.34^*^	0.44	0.11	2.08
Physician–assisted dying vs. suicide	−13.03	−24.02	−2.03	−0.44^*^			−22.73	−39.77	−5.69	−0.78^*^				
Violent death vs. non-violent death							10.31	−5.00	25.63	0.32				
Present at death vs. not present							18.54	−0.64	37.71	0.63				
**PTSD symptoms**														
Years since death	−0.88	−2.31	0.54	−0.27	1.33	0.10	−0.98	−2.38	0.43	−0.30	1.51	0.22	0.12	1.63
Physician-assisted dying vs. suicide	−4.97	−12.08	2.14	−0.31			−9.16	−20.38	2.06	−0.56				
Violent death vs. non-violent death							7.40	−2.68	17.48	0.42				
Present at death vs. not present							9.93	−2.69	22.55	0.61				
**Anxiety symptoms**														
Years since death	−0.05	−0.54	0.44	−0.04	2.01	0.14	−0.08	−0.58	0.41	−0.07	1.51	0.22	0.07	1.01
Physician-assisted dying vs. suicide	−2.27	−4.72	0.19	−0.39			−4.38	−8.35	−0.41	−0.76^*^				
Violent death vs. non-violent death							0.39	−3.17	3.96	0.06				
Present at death vs. not present							2.82	−1.65	7.29	0.49				
**Depression symptoms**														
Years since death	−0.33	−0.97	0.32	−0.22	0.70	0.05	−0.38	−1.01	0.25	−0.26	1.38	0.20	0.15	2.00
Physician-assisted dying vs. suicide	−1.47	−4.69	1.76	−0.20			−5.26	−10.28	−0.24	−0.73^*^				
Violent death vs. non-violent death							0.21	−4.30	4.72	0.03				
Present at death vs. not present							4.74	−0.91	10.39	0.66				
**General quality of life**														
Years since death	−0.01	−2.57	2.55	0.00	1.31	0.00	0.10	−2.55	2.75	0.02	0.22	0.04	0.04	0.44
Physician-assisted dying vs. suicide	0.60	−12.14	13.34	0.02			7.56	−13.57	28.68	0.27				
Violent death vs. non-violent death							−3.15	−22.12	15.83	−0.10				
Present at death vs. not present							−10.50	−34.27	13.26	−0.38				
**Functional impairment**														
Years since death	−0.23	−1.60	1.13	−0.08	0.75	0.06	−0.28	−1.68	1.12	−0.09	0.67	0.11	0.05	0.61
Physician-assisted dying vs. suicide	−3.99	−10.79	2.80	−0.26			−5.85	−17.03	5.33	−0.38				
Violent death vs. non-violent death							4.94	−5.11	14.98	0.30				
Present at death vs. not present							5.49	−7.09	18.07	0.36				

* *P < 0.05*.

** *P < 0.01*.

***P < 0.001.

## Discussion

Our study shows that death by PAD due to a mental disorder is associated with lower grief reactions in bereaved partners compared to death by suicide. A systematic review found that people bereaved by PAD scored similar or lower on disordered grief, mental health and post-traumatic stress compared to bereaved by natural causes (25), possibly related to the bereaved person's involvement in the decision-making process and being convinced the deceased chose death willingly. Our study shows that these latter aspects are not unique to PAD and occur in some cases of suicide as well. Bereaved partners could both respect and understand the feeling of their partner that life was no longer livable, while maintaining hope that their partner would change their mind about pursuing PAD or suicide. We found that a granted request for PAD was seen as validation of the severity of the suffering, both from the perspective of the bereaved as well as their social environment. A similar result was found in an interview study among patients with a PAD-request suffering from mental disorders, where patients were looking for recognition of their suffering (38). Expectedness of the death of the partner, lack of suffering of the partner at the time of dying, and presence of physician support may in part explain the protective effects of PAD against severe grief reactions.

### Finding the Right Words

The phrase “a wish to die” is often used in relation to PAD, but may not reflect the lived experience of not being able to continue living. For many bereaved partners, their loved one's death eventually was the only option, since their life was no longer bearable. This is in line with a previous study into considerations on PAD of patients suffering from a mental disorder (38). Furthermore, there is no consensus as to whether or not a suicide can be considered a choice. Some bereaved partners do see it as a choice, although they may perceive their loved one had no reasonable alternative options. While PAD and suicide sometimes seem to be understood as opposites, this does not adequately represent partner's perceptions. Indeed, some suicide-bereaved respondents used terms such as “self-euthanasia” or “gray zone” to describe their partner's suicide. Finally, the use of the terms violent and non-violent death may not match the experience of the bereaved. Being present at a non-violent, non-mutilating suicide can be experienced as violent.

Finding words that do justice to the experience of the bereaved is all the more important as bereaved partners can already feel isolated from others by feeling misunderstood, and it contributes to a feeling of being heard and can strengthen the relationship with others.

People who die by assisted suicide are more likely to be women, older, higher educated, widowed and living alone, compared to people who die by unassisted suicide (39). While we do not have information about the level of education of the deceased in our study, based on the level of education of their partners it is likely that the deceased by suicide in our study had been higher educated than the deceased by PAD. Age differences between the groups resemble findings in previous reports: while 69% of people who died by suicide motivated by a mental disorder is under 60 years of age and 13% is over 70 years of age, 56% of psychiatric patients who died by PAD is under 60 and 23% is over 70 years of age (11, 17). Differences in duration of the relationship are probably related to the age difference between the two groups.

### Study Strengths and Limitations

To our knowledge, this is the first study of the experience and grief of life partners of people who died by PAD or suicide due to a mental disorder. A strength of our study is including both mutilating as well as non-mutilating, “humane” suicide methods (40). The study had the following limitations. First, for the quantitative analyses, our sample size was small. Although a small sample size is generally associated with a low power to detect statistically significant associations, we found several significant associations to confirm our hypotheses, suggesting that our sample size had enough statistical power. However, generalizability of our findings may be limited by the small sample size. Second, generalizability may be limited due to self-selection of participants in the study. It could bias the results if people were motivated to participate because of negative experiences, thus over representing the severity of mental health problems. Conversely, if people were motivated by a desire to advocate for a specific method to die (particularly expected in PAD), this could lead to underrepresentation of severe mental health problems. Since the actual experiences of the participants were not one-sided–they also mentioned difficulties experienced in the process of PAD and positive aspects of suicide–we expected that self-selection bias did not skew our results. Third, our study took place in the Netherlands, and the results may not be generalizable to other jurisdictions. People from countries where PAD or PAD due to a mental disorder is not allowed may sometimes travel to other countries to request PAD. In such cases, PAD comes at the end of a difficult pathway where partners' feelings and grief may be differently affected from what this study shows. Fourth, our study took place during the Covid-19 pandemic, when social life was restricted due to lockdown. This might have influenced grief reactions, e.g., feelings of isolation.

### Implications for Practice and Further Research

The growing demand of PAD due to a mental disorder forces psychiatrists to position themselves toward these requests. More attention for PAD in mental health care is warranted (41). Physicians who consider personal involvement in PAD due to a mental disorder may take grief reactions of the bereaved partner into account when taking position on how to deal with PAD requests from psychiatric patients. Partners of people who consider to end their own life should receive professional support, and their concerns should be taken seriously. The guideline of the Dutch Association for Psychiatry states that involving loved ones during the process toward PAD due to a mental disorder is prudent (42), contributing to experiencing a joint trajectory. Such involvement may be desirable in all patients considering to end their life, to mitigate suicide risk and to support bereaved loved ones following eventual death.

## Data Availability Statement

The datasets presented in this article are not readily available because of the ethically sensitive nature of the research and potential participant identifiers in the datasets. Requests to access the datasets should be directed to m.snijdewind@centrum45.nl.

## Ethics Statement

Ethical review and approval was not required for the study on human participants in accordance with the local legislation and institutional requirements. The patients/participants provided their written informed consent to participate in this study.

## Author Contributions

MS carried out the interviews. GS and GC contributed to the data acquisition. Initial coding was done by MS and two assistants. MS and GS contributed to the analysis, interpretation of quantitative and qualitative data, and drafted the manuscript. All authors contributed substantially to the conception or design of the work, revised it critically for important intellectual content, and approved the final version to be published.

## Funding

This work was supported by ARQ Centrum'45, Vrienden van Expertisecentrum Euthanasie, and Stichting Stimuleringsfonds Rouw – a foundation stimulating attention for and knowledge development about grief.

## Conflict of Interest

The authors declare that the research was conducted in the absence of any commercial or financial relationships that could be construed as a potential conflict of interest.

## Publisher's Note

All claims expressed in this article are solely those of the authors and do not necessarily represent those of their affiliated organizations, or those of the publisher, the editors and the reviewers. Any product that may be evaluated in this article, or claim that may be made by its manufacturer, is not guaranteed or endorsed by the publisher.
