# Supplementary material for: “Only One Way Out”-Partners' Experiences and Grief Related to the Death of Their Loved One by Suicide or Physician-Assisted Dying Due to a Mental Disorder
Source: Front Psychiatry. 2022 Jul 8;13:894417. doi: 10.3389/fpsyt.2022.894417 (PMC9304962; doi:10.3389/fpsyt.2022.894417)
Supplement: Supplementary file 1 [file Data_Sheet_1.pdf]

Supplementary Material

1 Supplementary Table 1

Topic list (translated, original in Dutch)

Interview introduction	Information about the research project<br>Recording and confidentiality<br>Questions<br>Informed consent
Introductory questions	Reason(s) to participate<br>Name of the partner<br>General information about partner
Mental disorder of the deceased partner	First notion of metal disorder(s) and its impact<br>Good day/period of time<br>Bad day/period of time<br>Awareness/reaction social environment
Care providers	(Professional) care related to the mental disorder(s)
Potential/possible death of the partner	Possible death: awareness/topic of discussion/ suicidal thoughts or tendencies<br>Thoughts on ending one's life within received (professional) care
Passing of the partner and life afterwards	Way of dying, meaning for bereaved partner<br>Farewell<br>Period of time following death<br>Reactions social environment<br>Openness and sharing, support, loneliness
Current situation	Interview experience<br>Memories<br>Emotions<br>Influence loss on current life<br>Difficulties<br>Support and comfort<br>Openness social environment<br>View on death<br>Outlook on future
Ending	Recap<br>Other important experiences related to the loss?

2 Supplementary Table 2

Grief disorder symptoms by mode of partner's death

	Full sample		Mode of partner's death				
	(N = 27)		Suicide (N = 15)		PAD (N = 12)		
	N	%	N	%`	N	%	χ^2 (df = 1)
DSM-5 PCBD symptoms	32.59	10.37	34.57	9.86	30.13	10.88	1.11
DSM-5-TR PGD symptoms	23.02	8.05	23.57	8.47	22.33	7.80	0.39
ICD-11 PGD symptoms	26.32	9.28	27.48	9.32	24.86	9.42	0.73

* $p < .05$

** $p < .01$

3 Supplementary Table 3

Multiple regressions predicting grief disorder symptoms based on mode of partner's death and time since death

	Step 1					Step 2						
	B	95% CI	Beta	F	R^2	B	95% CI	Beta	F	R^2	ΔR^2	ΔF
DSM-5 PCBD symptoms				5.87 **	0.33				4.56 **	0.45	0.12	2.51
Years since death	-2.39	-3.94 -0.83	-0.58 **			-2.53	-4.01 -1.05	-0.61 **				
Physician-assisted dying vs. suicide	-9.24	-16.97 -1.51	-0.45 *			-16.71	-28.50 -4.93	-0.82 **				
Violent death vs. non-violent death						7.68	-2.91 18.27	0.34				
Present at death vs. not present						14.11	0.84 27.37	0.69 *				
DSM-5-TR PGD symptoms				9.71 ***	0.45				7.78 ***	0.59	0.14	3.68 *
Years since death	-2.32	-3.41 -1.23	-0.73 ***			-2.43	-3.43 -1.43	-0.76 ***				
Physician-assisted dying vs. suicide	-5.90	-11.34 -0.46	-0.37 *			-11.59	-19.55 -3.63	-0.73 **				
Violent death vs. non-violent death						6.78	-0.37 13.94	0.39				
Present at death vs. not present						11.36	2.40 20.31	0.71 *				
ICD-11 PGD symptoms				10.25 **	0.46				6.45 **	0.54	0.08	1.89
Years since death	-2.67	-3.92 -1.43	-0.73 ***			-2.77	-3.99 -1.56	-0.75 ***				
Physician-assisted dying vs. suicide	-8.00	-14.19 -1.80	-0.44 *			-13.27	-22.95 -3.60	-0.72 **				
Violent death vs. non-violent death						5.54	-3.15 14.23	0.28				
Present at death vs. not present						10.03	-0.85 20.92	0.55				

* $p < .05$

** $p < .01$

*** $p < .001$
